# Lipid nanoparticles for nucleic acid delivery to osteoblasts and osteoblast-lineage cells: A systematic review

**DOI:** 10.1016/j.bonr.2026.101921

**Published:** 2026-05-12

**Authors:** Alexander I. Murphy, Christopher Vargas, Jason H. Pomerantz

**Affiliations:** Departments of Surgery and Orofacial Sciences, Division of Plastic and Reconstructive Surgery, Program in Craniofacial Biology, Eli and Edythe Broad Center of Regeneration Medicine, University of California, San Francisco, 505 Parnassus Avenue, M-593, San Francisco, United States

**Keywords:** Lipid nanoparticles, Nucleic acids, Gene therapy, Bone, Osteoblast

## Abstract

**Background:**

Lipid nanoparticle (LNP) delivery of nucleic acids is a promising therapeutic platform for addressing bone disorders. A review of LNP formulations that have been studied for nucleic acid delivery to osteoblasts and osteoblast-lineage cells may help guide development of LNPs that are optimized for nucleic acid treatment of bone disorders.

**Methods:**

In October 2025, a search of PubMed, Embase, and Web of Science was conducted to systematically review studies on LNPs that have been evaluated for nucleic acid delivery to osteoblasts and osteoblast-lineage cells. Nine studies were included for analysis.

**Results:**

In the nine included studies, 11 LNPs were evaluated for nucleic acid delivery to osteoblasts or osteoblast-lineage cells. The LNPs were formulated with various ionizable/cationic lipids, helper lipids, PEGylated lipids, and bone-targeting ligands. They carried mRNA, siRNA, or long non-coding RNA cargos. All LNPs were capable of transfecting target cells with nucleic acids in vitro and/or delivering nucleic acids to bone tissues in rodent models. The delivery efficiency differed between LNPs with different formulations. Cytotoxicity of ionizable LNPs in cell cultures was minimal, and in vivo adverse effects were limited to elevated inflammatory markers for certain LNP formulations.

**Conclusions:**

This study catalogs 11 different LNP formulations that are effective for delivery of nucleic acids to bone cells in in vitro and/or in vivo models. Differences in performance and formulation of these LNPs can help inform optimization of LNP formulation for nucleic acid treatment of bone disorders.

## 1 Introduction

Nucleic acid-based drug delivery with lipid nanoparticles (LNPs) is a promising platform for disease prevention and treatment (Naeem et al., 2025). The therapeutic mechanism is determined by the drug's nucleic acid cargo, such as mRNA or siRNA, which are used to modulate intracellular expression of nucleic acids and proteins to affect disease processes. LNPs hold a number of advantages over other vector options to deliver nucleic acids to cells (Cullis and Felgner, 2024). LNPs do not integrate into host genomes, have low immunogenicity, and have properties that facilitate their manufacturing for large-scale distribution as pharmaceuticals (Geng et al., 2025). To date, there are three FDA - approved drugs that deploy LNPs to deliver nucleic acids, including Onpattro for treatment of hereditary transthyretin-mediated amyloidosis, the Moderna COVID-19 vaccine, and the Pfizer COVID-19 vaccine (Namiot et al., 2023). Success of these drugs provides strong support for developing approaches utilizing LNP delivery of nucleic acids to address other diseases.

Bone-related disorders are potentially treatable using this platform, but LNP delivery of nucleic acid to bone involves a long journey with many obstacles. After intravenous administration, the most common route of administration for clinically-used LNP-delivered treatments, LNPs must remain stable in the serum, evade uptake in the liver, enter bone tissue, navigate bone sinusoids to bone cells, have affinity (and potentially specificity) for bone cells of interest, and deliver their cargo across cell membranes (Thi et al., 2021; Marenzana and Arnett, 2013; Hosseini-Kharat et al., 2025; Gustafson et al., 2015; Wang et al., 2010; Mehta et al., 2023; Wei et al., 2024). To meet these challenges, LNPs hold an important advantage over other vectors in their ability to be modified to address physical and chemical requirements. The modifiable components of LNPs include ionizable/cationic lipids, helper lipids, cholesterol, PEGylated lipids, and bone-targeting ligands (Mehta et al., 2023) (Wei et al., 2024). Each of these components can be adjusted to affect LNP characteristics such as serum stability, immune recognition, and cellular uptake. Together, these opportunities for LNP modification make identifying effective LNP formulations for in vivo nucleic acid delivery to bone cells a feasible goal.

In this study, we systematically reviewed the literature on LNP formulations that have been evaluated for delivery of nucleic acids to bone cells, specifically osteoblasts and osteoblast-lineage cells. The primary goals of this review are 1) to identify existing LNP formulations capable of transfecting these cells with nucleic acids in vitro, 2) to identify existing LNPs with specificity toward bone tissue versus other tissue types, and 3) to identify LNP formulation characteristics that may be utilized by others to improve bone transfection efficiency and specificity.

## 2 Methods

**Fig. 1:**
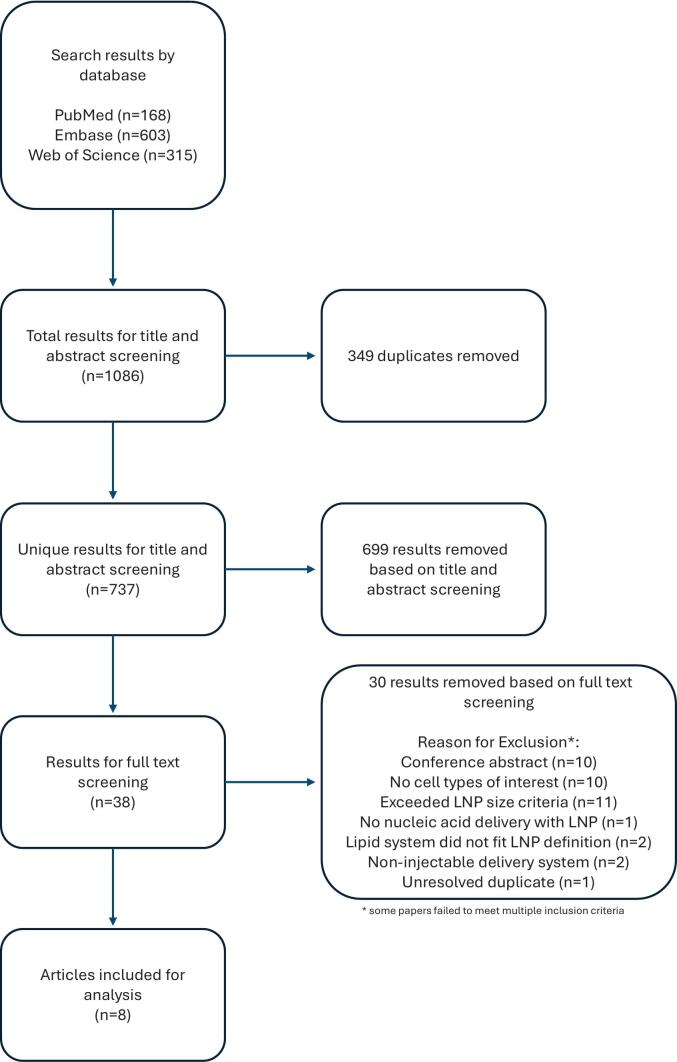
Preferred Reporting Items for Systematic Reviews and Meta-Analyses (PRISMA) flow chart depicting study inclusion and exclusion strategy.

Fig. 1 shows a Preferred Reporting Items for Systematic Reviews and Meta-Analyses (PRISMA) diagram depicting our systematic review process. In October 2025, a search was performed of PubMed, Embase, and Web of Science. The PubMed search utilized the following search string and yielded 230 results: (“lipid nanoparticles”[supplementary concept] OR “lipid nanoparticle*”) AND (“bone and bones”[mesh] OR “bone” OR “bones” OR “skeletal”). The Embase search utilized the following search string and yielded 759 results: (‘lipid nanoparticles’/exp. OR ‘lipid nanoparticle*’) AND (‘bone’/exp. OR ‘bone’ OR ‘bones’ OR ‘skeletal’). The Web of Science search utilized the following search string and yielded 397 results: (“lipid nanoparticle*”) AND (“bone” OR “bones” OR “skeletal”) (All Fields). A total of 1386 abstracts were exported to Rayyan (Qatar Computing Research Institute, Qatar), which was utilized to facilitate further review.

Two authors (AM and CV) then performed independent and blinded title and abstract screening to evaluate studies for inclusion. We specifically focused on injectable LNP formulations that deliver nucleic acids to osteoblasts and/or osteoblast-lineage cells in in vitro and/or in vivo models. Specific inclusion criteria are listed in Table 1. In an effort to include only lipidic delivery vectors that would widely be accepted as true LNPs but also not exclude LNPs with the potential for in vivo bone delivery, size inclusion criteria was based on both the NIH definition of nanomaterials (National Institutes of Health (NIH), 2026) and upper-limit estimates for bone sinusoid width (Wang et al., 2010). Conference abstracts, reviews, and articles not in English were excluded. Screening discrepancies were resolved by discussion between the two reviewers.

**Table 1:** Inclusion criteria.

1. Study evaluates lipid nanoparticles defined as: - Having lipidic formulations containing ionizable or cationic lipid; helper lipid; cholesterol; and PEGylated lipid. - Having an average diameter that is no more than one standard deviation greater than 100 nm. 2. Study includes lipid nanoparticle formulations capable of delivery of nucleic acids to osteoblasts and/or osteoblast-lineage cells in in vitro and/or in vivo models. 3. Formulation and route of treatment with lipid nanoparticles must be injectable.

Following resolution of duplicates, 926 articles underwent title and abstract screening, after which 49 articles were included for full text screening. An additional 40 papers were excluded by full text screening. Screening discrepancies were again resolved by discussion between authors. From the nine remaining studies, authors then extracted data including: specific treatment LNPs evaluated, constituent lipid formulations, nucleic acid cargo, average LNP diameters (with standard deviation when available), models for in vitro and in vivo analyses, primary results from these studies, and results from toxicity/adverse effects analyses.

To systematically evaluate risk-of-bias in our study, we utilized the Systematic Review Centre for Laboratory Animal Experimentation (SYRCLE) risk-of-bias tool (Hooijmans et al., 2014). Each study was evaluated by two authors (A.I.M. and C.V.) and disagreements in categories were resolved after discussion between these two authors.

## 3 Results

### 3.1 Included studies, LNP characteristics, and nucleic acid cargos

Nine studies were included, which described 11 different formulations that met study criteria as an LNP (Table 2). Various ionizable lipids were incorporated into the different LNPs. The most commonly used ionizable lipids (7/11 LNPs) were 1,2-dilinoleyloxy-N (DLin) – based, which is the ionizable lipid component of Onpattro (Akinc et al., 2019). Other ionizable lipids were used in LNPs in two studies. First, Zheng (2024) used ALC-0315 as their ionizable lipid (Zheng et al., 2024), which is the ionizable lipid used in the Pfizer COVID-19 vaccine (Zhang et al., 2023). Second, M. Li (2025) incorporated ionizable lipids A1I4R2C18-2 and A1I4R13C18-2 into their two LNPs ILR2-LNP and ILR13-LNP, respectively (Li et al., 2025a). These two ionizable lipids differ by only an ionizable R-group and were previously demonstrated capable of systemic in vivo delivery in mouse models (He et al., 2023). The only included LNP that did not incorporate an ionizable lipid is the CH6-LNP studied by Xu (2024), that instead incorporated 1,2-Dioleoyl-3-trimethylammonium-propane (DOTAP), a cationic lipid (Xu et al., 2024).

**Table 2:** Included studies, lipid nanoparticle characteristics, and cargo characteristics. Molar ratios listed as ionizable lipid: Helper Lipid: Cholesterol: PEGylated Lipid. *Abbreviations: LNP = lipid nanoparticle, SD* *= standard deviation, nm = nanometer, siRNA = small-interfering RNA, miRNA = micro RNA, pDNA = plasmid DNA, mRNA = messenger RNA.*

Study author and year	Treatment LNPs (Molar ratio, if provided)	Ionizable/cationic lipid	Helper lipid	PEGylated lipid	Bone-targeting ligand	Nucleic acid treatment cargo	Average LNP diameter (SD) in nanometers	Method for LNP diameter measurement
Liang et al., 2015	CH6-LNP	DLin-KC2-DMA	DPPC	DSPE-PEG2000-Mal	CH6 Aptamer	siRNA targeting *Plekho1*, with and without Cy3 label	84.0 (5.3)	Laser light scattering
Basha et al., 2016	**LNP** (50:10:38.5:1.5)	DLin-MC4-DMA	DSPC	DMG-PEG	–	- Quasar-570-labeled siRNA - siRNA targeting *Sclerostin*	36.93 (SD not stated)	Dynamic light scattering
Basha et al., 2022	**DMG-PEG LNP** (50:10:38.5:1.5)	DLin-MC3-DMA	DSPC	DMG-PEG	–	- siRNA targeting *GNAS* (with or without 2′ *O*-methyl bases) - Various fluorescent-labeled siRNAs - siRNA targeting *GFP* - siRNA targeting *Luciferase*	- With siLuc: 52.5 (SD not stated)	Dynamic light scattering
	**DSG-PEG LNP** (50:10:38.5:1.5)	D-Lin-MC3-DMA	DSPC	DSG-PEG	–			
							- With siGNAS: 52.0 (SD not stated)	
Liu et al., 2024	Zoledronic Acid Bone targeting LNP (50:10:38.5:1.5)	DLin-MC3-DMA	DSPC	DMG-PEG20000	Zoledronic Acid	- *GFP* mRNA - Luciferase mRNA - m7G methylated *Runx2* mRNA	- With *GFP* mRNA: 71.75 (16.54) - With Luc mRNA: 70.75 (15.74) - With Runx2 mRNA: 71.25 (16.68)	Dynamic light scattering
Xu et al., 2024	**CH6-LNP** (22.2:44.4:22.2:11.1)	DOTAP	DSPC	DSPE-PEG-MAL	CH6 Aptamer	siRNA for *NLRP3*	- With NLRP3 siRNA: 96.64 (16.83)	Dynamic light scattering
Nelson et al., 2024	**MC3 LNP** (50:10.5:38:1.5)	DLin-MC3-DMA	DSPC	DMG-PEG2000	–	- Luciferase mRNA - B-catenin mRNA	101.04 (1.97)	Dynamic light scattering
Zheng et al., 2024	**LNP** (52.7:21.7:21.4:4.1)	ALC-0315	DSPC	DMG-PEG2000	–	- *GFP* mRNA - *Usp26* mRNA	Fig. 1D demonstrates mean size <100 nm (SD not stated)	Dynamic light scattering
M. Li et al., 2025a	**ILR2-LNP** (50:10:38.5:1.5)	A1I4R22C18–2 R2	DSPC	DMG-PEG2000	–	- GAS5 lncRNA	63 (3)	Dynamic light scattering
	**ILR13-LNP** (50:10:38.5:1.5)	A1I4R22C18–2 R13	DSPC	DMG-PEG2000	–		80 (1)	
Z. Li et al., 2025b	**LNP@apt** (50:10:38.5:1.5)	D-Lin-MC3- DMA	DSPC	DMG-PEG-200	BMSC specific aptamer	- *EGFP* mRNA - *Pcolce* mRNA	Figure 4D demonstrates mean size <100 nm (SD not stated)	Dynamic light scattering

Two helper lipids were also incorporated into the different LNPs. Distearoylphosphatidylcholine (DSPC), another component of the Moderna COVID-19 vaccine (A Comprehensive List of COVID-19 Vaccine Ingredients, 2021), was the most commonly incorporated helper lipid (10/11 LNPs). Dipalmitoylphosphatidylcholine (DPPC) was the other helper lipid incorporated into one LNP.

For PEGylated-lipids, variants of myristoyl diglyceride-polyethylene glycol (DMG-PEG), another Onpattro component (Akinc et al., 2019), were the most commonly incorporated (8/11 LNPs), followed by 1,2-distearoyl-sn-glycero-3-phosphoethanolamine-N[amino-PEG (DSPE-PEG) (2/12 LNPs). Basha (2022) incorporated distearoyl-rac-glycerol-PEG (DSG-PEG) into one of their LNPs, hypothesizing that this PEGylated lipid may result in longer LNP circulation times compared to DMG-PEG, which may allow better delivery to osteoblasts in compact bone (Basha et al., 2022).

Four studies incorporated a bone-targeting ligand into their LNP formulations. Using a cell-SELEX assay to evaluate the affinity of a library of aptamers for primary rat osteoblasts, Liang (2015) identified aptamer CH6 as one with strong osteoblast binding ability and low affinity for rat liver or blood cells (Liang et al., 2015). Both Liang et al. (2015) and Xu et al. (2024) used this aptamer as the bone-targeting ligand for their LNPs. Z. Li et al. (2025b) also incorporated an aptamer into their LNP@apt, but instead this aptamer was previously optimized for binding to mouse bone-marrow mesenchymal stem cells (MSCs) (Li et al., 2025b). Liu (2024) evaluated zoledronic acid, a commonly used osteoporosis medication with high affinity for calcium, as their bone-targeting ligand (Liu et al., 2024).

LNPs were loaded with three different classes of nucleic acid treatment cargo. Four studies evaluated siRNA delivery, four studies evaluated mRNA delivery, and one study evaluated delivery of a long non-coding RNA (lncRNA). All cargos were either intended to activate a fluorescent/luminescent reporter to demonstrate transfection of cells or were intended to affect expression of a protein involved in bone anabolism.

The average diameter of each LNP varied greatly. The smallest LNP was the DLin-MC4-based LNP studied by Basha et al. (2016) (36.93 nm). The largest LNP was the MC3 LNP from the Nelson et al. (2024) study. Of note, in addition to the LNPs that were included in Table 2, two studies also evaluated additional lipidic delivery vectors that did not meet the size criterion for inclusion in Table 2. In addition to their MC3 LNP, Nelson et al. (2024) described a lipidic delivery vector incorporating an SM102 ionizable lipid, but this vector's size was higher than the size inclusion criteria for our study. In addition to their ILR2-LNP and ILR13-LNP, M. Li et al. (2025a) evaluated three additional ionizable lipid A1I4RnC18-2-based lipidic delivery vectors, but each of the three were also too large to meet inclusion criteria for our study.

### 3.2 In vitro studies

Table 3 summarizes the primary results and measures of LNP toxicity for the different in vitro cell models in the included studies.

**Table 3:** In vitro models, results, and toxicity studies. *Abbreviations: LNP = Lipid nanoparticle, MTT = (3-[4,5-dimethylthiazol-2-yl]-2,5 diphenyl tetrazolium bromide), PBS = phosphate buffered saline, SD = Sprague-Dailey, miRNA = micro RNA, siRNA = small interfering RNA, mRNA = messenger RNA, WT = wild-type, ROS = reactive oxygen species, pDNA = plasmid DNA.*

Study author and year	Models	Primary results	Adverse effects
Liang et al., 2015	Rat primary osteoblasts	- CH6-LNPs delivered *Plekho* siRNA to osteoblasts at higher efficiency and produced higher knockdown efficiency than delivery with random aptamer LNPs and aptamer-free LNPs. There was a dose response for higher concentrations of siRNA, with average knockdown efficiency of approximately 80% for the highest siRNA concentration tested (80 nM) compared to untreated controls. - CH6-LNPs entered osteoblasts through micropinocytosis and clathrin-mediated endocytosis.	No cytotoxicity noted for treatment with CH6-LNP using MTT assay.
Basha et al., 2016	Primary mouse embryonic fibroblasts induced to osteocyte phenotype using osteogenic medium	- LNPs more efficiently delivered fluorescent-siRNA and siRNA targeting *Sclerostin* compared to free siRNA. Delivery efficiency was nearly four times higher with LNPs than free siRNA. - *Sclerostin* siRNA delivered with LNP induced knockdown of *Sclerostin* mRNA in dose dependent manner and promoted osteogenic differentiation.	Cell toxicity was not seen on microscopy over the 15 days following LNP-*Sclerostin* siRNA administration.
Basha et al., 2022	**-** Mouse embryonic fibroblasts (“mesenchymal stem cell-like cells”) induced with osteogenic media for *GNAS* knockdown, differentiation, and toxicity studies - Primary bone-derived mesenchymal stem cells of *GFP* transgenic mice for transfection efficiency and *GFP* knockdown studies	- Delivery of *GNAS* siRNA with both PEG-DMG and PEG-DSG LNPs led to more efficient mRNA knockdown than PBS control, and PEG-DMG LNP delivery led to more potent knockdown (70–80%) than PEG-DSG LNP (50–60%). - PEG-DMG LNP-delivered *GNAS* siRNA induces upregulation of osteogenic markers and osteogenic differentiation, with longer-effects than LNP-mediated control siRNA delivery. PEG-DSG LNP was not tested. - PEG-DSG LNP efficiently transfected 70–80% of mesenchymal stem cells with TYE 563-labeled siRNA and induced dose dependent knockdown of *GFP* mRNA when delivering *GFP* siRNA versus negative controls. PEG-DMG LNP was not tested.	No evidence of toxicity on microscopic evaluation of cells over 15 days after PEG-DMG siRNA delivery.
Liu et al., 2024	Mouse bone marrow mesenchymal stem cells	- Zoledronic Acid-LNP loaded with *GFP* mRNA delivered *GFP* mRNA and induced higher expression compared to unnamed negative controls.	–
Xu et al., 2024	Ovariectomized and non-ovariectomized rat osteoblasts	- *NLRP3* siRNA delivered with CH6-LNPs and Lipofectamine-3000 control similarly reduced the expression (approximately 50% knockdown) of *NLRP3* and increased osteogenesis versus free *NLRP3* siRNA.	No cytotoxicity noted for any LNP treatments, as determined by MTT assay.
Nelson et al., 2024	Human bone marrow mesenchymal stem cells	- MC3 LNP delivery of *luciferase* mRNA resulted in lower luciferase luminescence than delivery with an SM102 formulation, which were both significantly higher than PBS control. The luminescence was nearly four times as high in the SM102 treated cells.	Neither LNP produced cytotoxic effects as measured by Celltiter Blue Reagent.
Zheng et al., 2024	Rat bone marrow mesenchymal stem cells	- LNPs loaded with *GFP* mRNA transfected cells at higher efficiency than unnamed control. LNP transfection efficiency was 99.84%. - Treatment with *Usp26* mRNA-loaded LNPs increased *Usp26* mRNA levels, osteogenic differentiation markers, and mineralization compared to unnamed control.	Treatment with gel microspheres containing LNPs did not induce cytotoxicity as measured by live/dead staining.
M. Li et al., 2025a	Rat bone marrow mesenchymal stem cells	- Treatment with ILR2-LNP and ILR13-LNP loaded with GAS5 lncRNA resulted in greater intra-cellular GAS5 lncRNA and osteogenic differentiation compared to an unnamed control. - Treatment with ILR2-LNP and ILR13-LNP loaded with GAS5 lncRNA resulted in approximately half as much intra-cellular GAS5 lncRNA compared to delivery of a similar formulation with larger size and a hydrophilic hydroxy linker group.	–
Z. Li et al., 2025b	Mouse bone marrow mesenchymal stem cells	- Treatment with LNP@apt loaded with *EGFP* mRNA resulted in twofold greater EGFP mRNA in cells compared to delivery with LNP without aptamer. - Treatment with LNP@apt loaded with *PCOLCE* mRNA resulted in *PCOLCE* mRNA in cells, as well as significantly increased *PCOLCE* protein expression relative to treatment with an LNP alone and the aptamer-less LNP loaded with *PCOLCE* mRNA	No significant cytotoxicity noted in Cell Counting Kit-8 assay in mouse bone marrow mesenchymal stem cells over three days Hemolysis testing resulted in less than 5% hemolysis at various LNP concentrations

#### 3.2.1 Mouse embryonic fibroblasts induced to osteocyte phenotype

Two studies used an in vitro model of mouse embryonic fibroblasts induced to an osteocyte phenotype with osteogenic medium. Basha et al. (2016) used this model to evaluate their DLin-MC4-based LNP's ability to deliver siRNA targeting *Sclerostin*, an inhibitor of osteogenic differentiation. They first evaluated transfection efficiency in these cells using fluorescently labeled *Sclerostin* siRNA and showed increased fluorescent intensity when siRNA was delivered with their LNP versus as free siRNA. Next, they compared mRNA knockdown efficiency of *Sclerostin* siRNA when it was delivered with LNPs versus as free siRNA, showing that the mRNA knockdown was greater for LNP-delivered siRNA. This increased knockdown was also associated with greater promotion of osteogenic differentiation of the cells. They did not compare this LNP to other delivery vectors.

In their study using the same cell line, Basha et al. (2022) first evaluated the ability of siRNA targeting *GNAS*, another inhibitor of osteogenic differentiation, to induce *GNAS* knockdown when delivered with their DMG-PEG and DSG-PEG LNPs. Similarly, they found improved knockdown with both LNPs versus negative controls, and they also saw improved knockdown with DMG-PEG relative to DSG-PEG.

#### 3.2.2 Rat primary osteoblasts

Two studies transfected rat primary osteoblasts in vitro. Liang et al. (2015) first evaluated transfection efficiency of fluorescently labeled siRNA delivered with their CH6-LNP and found increased cellular fluorescence compared to delivery with LNPs with random aptamers and aptamer-free LNPs. They then used CH6-LNPs to deliver siRNA targeting *Plekho*, a negative regulator of bone formation, and found more efficient knockdown of *Plekho* when delivered with CH6-LNP compared to delivery with the same controls. In the same model, Xu also studied CH6-LNP delivery of siRNA but instead with siRNA targeting *NLRP3*, a component of an inflammasome that promotes osteoporosis. They found improved knockdown with CH6-LNP-delivered *NLRP3* siRNA versus free *NLRP3* siRNA. Additionally, CH6-LNP delivery produced similar knockdown levels versus delivery with Lipofectamine (Thermofisher, Waltham, MA), a commonly used lipid-based transfection reagent.

#### 3.2.3 Mesenchymal stem cells

Six studies used various MSC models for their in vitro analyses. Using human bone-marrow MSCs, Nelson et al. (2024) compared delivery of *Luciferase* mRNA with their MC3 LNP versus the SM102 formulation and a PBS control. They found MC3 delivery resulted in higher luminescence than PBS but lower than delivery with the SM102 formulation.

In mouse bone-marrow MSCs, Liu et al. (2024) found that their Zoledronic Acid-LNP successfully delivered *GFP* mRNA at higher efficiency than negative controls, but they did not state what were used as controls. Also in mouse bone-marrow MSCs, Z. Li et al. (2025b) studied delivery of both mRNA for *GFP* and *Pcolce*, an enhancer of bone collagen formation. They found delivery with their LNP@apt resulted in higher cellular expression of both proteins for both mRNAs compared to delivery with an aptamer-less control. In their 2022 study, Basha et al. (2022) evaluated DSG-PEG LNP delivery of *GFP* siRNA to bone-derived MSCs from a transgenic *GFP*-expressing mouse and found a greater reduction in *GFP* fluorescence versus PBS controls. They did not evaluate DMG-PEG LNP in this model.

Zheng et al. (2024) used rat bone marrow MSCs to evaluate delivery of mRNA for *GFP* and *Usp26*, a protease that is a positive regulator of osteoblast differentiation. Compared to unnamed negative controls, they found that LNP-delivered *GFP* mRNA resulted in a higher GFP signal, and LNP-delivered *Usp26* mRNA resulted in higher Usp26 expression. M. Li et al. (2025a) also used rat bone marrow MSCs to study delivery of *GAS5*, a lncRNA capable of inducing differentiation of MSCs into osteoblasts. They found that delivery of *GAS5* with both ILR2-LNP and ILR13-LNP resulted in significantly higher cellular GAS5 versus an unnamed control. However, delivery of GAS5 was not as efficient with these two LNPs compared to a larger lipidic vector with a hydrophilic hydroxy R-group.

#### 3.2.4 In vitro toxicity

Seven studies also assessed various measures of in vitro LNP toxicity. Liang et al. (2015) and Xu et al. (2024) both evaluated their CH6 LNPs using a 3-(4,5-di methyl thiazol-2-yl)-2,5-diphenyltetrazolium bromide (MTT) colorimetric assay, and the authors reported that neither LNP resulted in any signs of cellular toxicity. In both their 2016 and 2022 studies, Basha treated their cells with siRNA delivered by each of their LNPs and noted cells did not show an appearance of cell injury on direct microscopic analysis. Nelson et al. (2024) noted no signs of toxicity for MC3 LNP in a CellTiter-Blue fluorometric cell viability assay. Zheng et al. (2024) did not identify any signs of toxicity in their live/dead staining assays after treatment with their LNP formulation. Z. Li et al. (2025b) found no significant cytotoxicity of their LNPs using a Cell Counting Kit-8 assay, and they also demonstrated less than 5% hemolysis at various LNP concentrations.

### 3.3 In vivo studies

Eight studies also evaluated the included LNPs in vivo using either rat or mouse models (Table 4).

**Table 4:** In vivo models, results, and adverse effects. *Abbreviations: siRNA = small-interfering RNA, mRNA = messenger RNA, LNP = lipid nanoparticle, PBS = phosphate-buffered saline, TNFa = Tumor Necrosis Factor alpha, CRP = C-reactive protein.*

Study author and year	Study design and models (Route of in vivo LNP administration)	Primary results	Study demonstrated direct delivery to bone tissue?	Adverse effects
Liang et al., 2015	Healthy SD rats and ovariectomized SD rats (tail vein injection)	- CH6-LNPs showed preferential siRNA delivery to bone tissue and osteoblasts versus other non-bone tissues and cell types. For CH6-LNP, the mean percentage of injection dose delivered to bone (versus other tissues) was approximately 10% compared <5% delivery to bone with random aptamer LNPs and LNPs without aptamers. - CH6-LNP-delivered *Plekho* siRNA induced stronger *Plekho* mRNA knockdown in osteoblasts versus other cell types, and weaker off-target delivery when compared to random aptamer and aptamer-free LNPs. - CH6-LNP-*Plekho* siRNA treatment improved bone structure and markers of bone anabolism in ovariectomized rats when compared to random aptamer LNPs.	Yes	Rats showed no liver/kidney toxicity or elevated serum immune markers relative to PBS-injected rats.
Basha et al., 2016	- C57Bl/6-GFP for biodistribution studies - Wild-type C57Bl/6 mice for knockdown studies (tail vein injection for all models)	- LNP successfully delivered fluorescent-siRNA to osteocytes in mouse tibia as visualized on fluorescent microscopy. The average proportion of osteocytes with fluorescent siRNA signal was approximately 50%. - LNP delivery of *Sclerostin* siRNA reduced sclerostin serum protein and tibial mRNA levels compared to treatment with LNP-delivered random siRNA sequence	Yes	No weight loss was seen in mice receiving LNP-siRNA treatments.
Basha et al., 2022	- Wild-type C57Bl/6 mice - C57Bl/6-GFP mice (tail vein injection for all models)	- PEG-DSG LNP delivered Dil-labeled siRNA to compact femur bone devoid of bone marrow with significant overlap with *GFP* expression in MSCs. PEG-DMG LNP was not tested. - PEG-DSG delivered DiO-labeled siRNA to approximately 70% of MSCs in femur compared to PBS controls. PEG-DMG LNP was not tested. - PEG-DSG delivery of *GFP* siRNA significantly reduced *GFP* expression versus PEG-DSG delivery of control RNA in mesenchymal stem cells from compact bone. PEG-DMG LNP not tested. - PEG-DSG delivery of *GNAS* siRNA significantly reduced *GNAS* expression and expression of osteogenic markers compared to control siRNA. PEG-DMG LNP was not tested.	Yes for PEG-DSG PEG-DMG not tested	No weight loss or signs of toxicity were noted when PEG-DSG LNP-siRNA was delivered.
Liu et al., 2024	- 2 month-old C57BL/6 mice via for luciferase study (via tail vein injection) - C57BL/6 mice with femoral holes for bone healing study (via direct injection into bone defect) - 12 month-old C57BL/6 mice for bone mass study (tail vein injection)	- Zoledronic Acid-LNP loaded with *luciferase* mRNA induced high luminescence in femur versus non-bone tissue (kidney, liver, spleen, heart, lung). - Zoledronic Acid-LNP loaded with methylated *Runx2* mRNA induced higher bone repair in femoral defects relative to untreated control mice. - Zoledronic Acid-LNP loaded with methylated *Runx2* mRNA increased bone mass in distal femur metaphysis relative to untreated control mice.	Yes	–
Xu et al., 2024	- Healthy SD rats for biodistribution studies - Ovariectomized SD rats for therapeutic studies (tail vein injection for all models)	- Compared to LNP without CH6 and negative controls, CH6-LNPs delivered Cy3-labeled *NLRP3* siRNA more efficiently to bone and less efficiently to liver and kidney without delivery to heart, lung, spleens. Mean fluorescent intensity in the femur was nearly eight times higher for the CH6-LNP at 24 h. - CH6-LNP delivery of *NLPR3* siRNA improved bone mass and architecture and increased markers of osteogenesis compared to aptamer-less LNP delivery.	Yes	No liver/kidney toxicity was noted on histology following CH6-LNPs-siNLRP3 administration, with low hemolysis on blood cell assay.
Nelson et al., 2024	C57BL/6 J mice with mid-shaft tibia fractures (local fracture site injection)	- MC3 LNP delivery of *Luciferase* mRNA resulted in higher luminescence at fracture site versus PBS control. Luminescence was similar compared with SM102 lipidic delivery vector formulation. Biodistribution did not differ from controls at other sites including liver, lung, kidney, and spleen.	Yes	Treatment with MC3 LNP led to increases in TNFα and CRP.
Zheng et al., 2024	Ovariectomized intervertebral fusion rats (local vertebral fusion site injection)	- Treatment with gel microspheres containing *Usp26* mRNA loaded-LNPs led to increased intervertebral bone generation and mineralization relative to non-microsphere *Usp26* mRNA LNPs.	No	–
M. Li et al., 2025a	–	–	–	–
Z. Li et al., 2025b	-Healthy C57BL/6 J mice (tail vein injection) -Ovariectomized C57BL/6 J mice (tail vein injection)	- Treatment with DiR-labeled LNP@apt resulted in higher fluorescent signal in bone compared to aptamer-less DiR-labeled LNP. Fluorescent signal was 2-4× higher in the legs and nearly 10× higher in the spine. -LNP@apt delivery of *PCOLCE* mRNA in ovariectomized mice resulted in improved bone microarchitecture, higher mineral deposition, and increased bone density versus untreated control	Yes	Histological analysis demonstrated normal tissue morphology and absence of inflammatory lesions in LNP@apt treated mice

#### 3.3.1 Rat models

The most commonly used rat model was the Sprague Dawley (SD) rat treated via tail vein injection. Liang et al. (2015) administered CH6-delivered fluorescently labeled siRNA in this model and found that fluorescent signal was higher in rat femur than in various organs including the heart, liver, spleen, lung, and kidney. Fluorescence in the liver after CH6 delivery was also less than delivery with an (Asp-Ser-Ser)_6_ liposome, suggesting the CH6-LNP had greater specificity for bone. Compared to aptamer-less LNP and random-aptamer LNP delivery, CH6-LNP delivery also resulted in relatively higher co-localization of fluorescence in areas stained with osteoblast markers, suggesting the CH6 aptamer resulted in improved osteoblast delivery in vivo. Compared to the same LNP controls, CH6-LNPs also produced reduced co-localization of fluorescent siRNA and markers for hepatocytes, Kupffer cells, and peripheral blood mononuclear cells versus these controls. After treatment with CH6-delivered *Plekho* siRNA, the authors then sorted for osteoblasts and found reduced *Plekho* mRNA expression in osteoblasts compared to delivery with the same controls. This treatment also resulted in increased signs of bone anabolism in ovariectomized SD rats compared to control LNPs.

Using their cationic CH6-LNP, Xu et al. (2024) performed similar studies in SD rats. After treatment with fluorescently labeled siRNA delivered with their CH6-LNP, fluorescent expression in bone was higher than in liver, kidney, heart, lung and spleen. In ovariectomized SD rats, Xu et al. (2024) also found that CH6-LNP delivery of *NLRP3* siRNA improved bone mass, bone architecture, and markers of osteogenesis compared to delivery with an aptamer-less control LNP. However, this study did not directly analyze in vivo delivery to osteoblast-lineage cells.

Zheng et al. (2024) also utilized an ovariectomized rat model, but their treatments were performed as local vertebral fusion site injections rather than intravenous injections. The authors injected LNPs carrying *Usp26* mRNA at these sites as two different compounds: as a standard LNP suspension and as LNPs incorporated into gel microspheres, which were hypothesized to prevent leakage from injection site and increase bioavailability. After injections of each compound into different rats, they compared intervertebral bone generation and mineralization and found the microspheres to be superior in both categories. This study neither evaluated direct in vivo delivery of nucleic acids to bone tissue nor to osteoblast lineage cells.

#### 3.3.2 Mouse models

C57Bl/6 mice treated via tail vein injection were the most commonly used murine model. Basha et al. (2016) injected red fluorescent-labeled LNP-siRNA into C57/Bl6 mice expressing *GFP* and identified consistent co-localization of the two dyes within osteocyte shaped structures in mouse tibia sections under confocal microscopy, suggesting LNP uptake in these bone cells. They also quantified this co-localization and concluded that about 50% of osteocytes had been transfected with LNP-siRNA. In a wild-type C57Bl/6 mouse, treatment with LNP-delivered *Sclerostin* siRNA led to decreased *Sclerostin* expression in bone tissue versus treatment with LNP-delivered random siRNA.

Basha et al. (2022) used tail vein injection of wild-type C57Bl/6 mice to quantify delivery of fluorescently labeled DSG-PEG LNP-siRNA to MSCs by sorting for MSCs in femur compact bone tissue and found nearly 70% uptake in these cells. They also used wild-type and *GFP*-expressing C57Bl/6 mice models to quantify knockdown of *GNAS* and *GFP* mRNA, respectively, after DSG-PEG LNP delivery of siRNA targeting the two proteins. In this experiment, the authors found increased knockdown in femur compact bone tissue for both treatments compared to LNP delivery of control siRNA. In the wild-type mouse model, they found this *GNAS* knockdown occurred in tibia/femur compact bone MSCs, as well as spleen, liver, and bone marrow cells. This knockdown correlated with decreased expression of osteogenic markers in bone. DMG-PEG LNP was not evaluated in in vivo studies.

In their 2-month-old C57Bl/6 mouse tail vein injection model, Liu et al. (2024) first injected Zoledronic Acid-LNP loaded with luciferase mRNA and found high luciferase signal in femur compared to kidney, liver, spleen, heart, and lungs. Next, in 12-month-old C57Bl/6 mice, a model that they use for senile osteoporosis, they performed tail vein injection of Zoledronic Acid-LNP loaded with mRNA for *Runx2*, a positive regulator of osteoblast differentiation, and found increased bone mass relative to untreated controls.

Using tail vein injections in healthy C57Bl/6 J mice, Z. Li et al. (2025b) administered fluorescently labeled LNP@apt and aptamer-less LNP and found higher fluorescent signal in bone for LNP@apt. They also treated an ovariectomized mouse with LNP@apt carrying *Pcolce* mRNA and showed improved signs of bone anabolism compared to untreated controls.

Two studies also evaluated LNP delivery of nucleic acids to bone when applied locally in bone injury models of C57Bl/6 mice. Liu et al. (2024) created femoral holes in their mice and locally applied Zoledronic Acid-LNP loaded with *Runx2* mRNA. They found improved repair of femoral holes relative to untreated control mice. Nelson et al. (2024) induced mid-shaft tibial fractures and delivered luciferase mRNA to the fracture sites with both MC3 LNP and the SM102 lipidic delivery vector. While luciferase signal was higher at the fracture site for both formulations versus a PBS control, the signal was similar to the control in other solid organs.

#### 3.3.3 In vivo toxicity studies

In vivo toxicity was also assessed in seven of the studies. Liang et al. (2015) showed administration of their CH6-LNP resulted in no liver or kidney toxicity and no difference in immune markers versus PBS injection. This was also true for Xu et al.'s (2024) CH6-LNP, that induced very low levels of hemolysis after intravenous injection. In their 2016 and 2022 studies, Basha reported no significant weight loss in mice treated with their 2016 LNP treatment or DSG-PEG, respectively. Nelson et al. (2024) evaluated changes in mouse inflammatory markers and found that MC3 LNP treatment resulted in elevations in TNF-alpha and CRP. Finally, Z. Li et al. (2025b) performed histological analysis of multiple solid organs in LNP@apt-treated mice, which showed normal tissue morphology and absence of inflammatory lesions.

#### 3.3.4 SYRCLE risk-of-bias evaluation

**Fig. 2:**
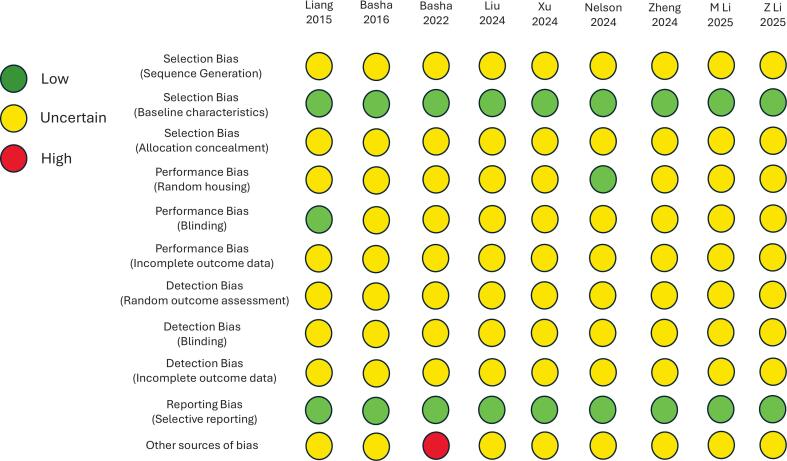
Systematic Review Center for Laboratory Animal Experimentation (SYRCLE) risk of bias tool diagram depicting risk of bias for included studies.

Fig. 2 displays the consensus results for the SYRCLE risk-of-bias assessment tool for each of the studies. Overall, most questions were answered as “uncertain risk-of-bias” due to the studies' lack of information required to assign risk using this tool. Of note, all studies reported similar baseline animal characteristics between treatment and control groups, making all studies low risk for this selection bias category. Similarly, selective reporting was not present in any study, also making each study low-risk for reporting bias. For the Basha et al. (2022) study, the authors utilize a device for LNP formulation manufactured by a company in which one of the authors has financial interest, which qualifies as high-risk for risk-of-bias according to SYRCLE.

## 4 Discussion

This systematic review identified 11 existing LNP formulations capable of delivering nucleic acids to osteoblast and/or osteoblast-lineage cells in vitro. Of these 11 LNPs, seven were clearly demonstrated to be capable of in vivo delivery of nucleic acids to bone tissue in rodent models. Herein, we discuss how the formulations of these LNPs may impact transfection of osteoblast-lineage cells and bone tissue, as well as which components of these LNPs may be harnessed to further optimize LNPs for this purpose moving forward.

In each of the included studies, the authors first evaluated the ability of LNPs to transfect osteoblasts or osteoblast precursors in vitro. Although most of the components of these LNPs were previously optimized for delivery to tissue types other than bone (Akinc et al., 2019; Zhang et al., 2023; He et al., 2023; Nelson et al., 2024), each LNP was indeed able to deliver nucleic acid to cultured osteoblast lineage cells. This finding demonstrates the broad potential of LNPs as drug delivery systems to a wide variety of cell types. Various controls were used to demonstrate this baseline ability of the LNPs to transfect cells of interest, and three of these studies compared LNP delivery of nucleic acids versus delivery with free nucleic acids (Xu et al., 2024; Basha et al., 2022; Basha et al., 2016). Unsurprisingly, LNP delivery transported nucleic acids into cells more efficiently in all cases. The passage of free nucleic acids across cell membranes is limited by their size and hydrophilic properties (Pei, 2022), and the findings summarized here demonstrate that LNPs increase delivery efficiency of nucleic acids to osteoblast-lineage cells in vitro models, regardless of the LNP's formulation.

After establishing that LNPs can transfect bone cells, some studies also compared in vitro transfection and knockdown efficiency of nucleic acids delivered with LNPs with differing ionizable lipid components. Ionizable lipids are one of the four principal components of LNPs formulations (Mehta et al., 2023). Ionizable lipids enable nucleic acid packaging, fusion of LNPs with target cell membranes, intracellular uptake of LNPs into endosomes, and ultimately escape of LNPs from endosomes to deliver nucleic acids to the cytosol (Han et al., 2021). Nelson (2024) compared delivery by their MC3 LNP with a larger lipidic delivery vector containing the ionizable lipid SM102 and found higher delivery efficiency with the SM102 formulation (Nelson et al., 2024). Previously, Hasset et al. compared the transfection efficiencies of SM102 and DLin-MC3-DMA LNPs in an in vivo intramuscular vaccine model and also found SM102 LNP treatment to result in higher cargo mRNA expression (Hassett et al., 2019). The authors attributed this difference to the relatively higher pKa of SM102, which may facilitate improved endosomal escape for mRNA delivery to cytosol. Although most LNPs identified in this review utilized DLin-based ionizable lipids, findings from our review suggest that SM102 should be considered for use in LNP formulations in future studies on nucleic acid delivery to bone cells.

Helper lipids are another LNP component that play an important role in LNP function. Several helper lipids are associated with increased stability of LNPs in serum, preventing their dissociation and facilitating delivery to distant tissues, while other helper lipids dissociate more easily, which facilitates endosomal escape (Cheng and Lee, 2016). Nelson's (2024) MC3 LNP and SM102 formulation also differed by their helper lipids, as the SM102 formulation contained DOPC and MC3 LNP contained DSPC (Nelson et al., 2024). DOPC and DSPC are often included in LNP formulations for their relatively strong abilities to stabilize LNPs and prevent serum degradation in vivo compared to other helper lipids (Barbieri et al., 2024). However, this stability also may impair endosomal escape, particularly for DSPC, and this provides another explanation for the lower in vitro transfection efficiency by the MC3 LNP. Thus, our review suggests that investigators must balance the relative stabilities of different helper lipids like DOPC and DSPC when formulating LNPs for in vitro delivery to bone cells versus for in vivo serum stability.

PEGylated lipids are the least abundant lipid components in LNP formulations but are important for LNP function. Along with an essential role in protecting LNPs from immune recognition (Tenchov et al., 2023), PEGylated lipids have also been shown to influence serum stability and transfection efficiency (Fang et al., 2017). Basha (2022) compared siRNA knockdown efficiency after delivery with two different LNPs that differed in their PEGylated lipid component (Basha et al., 2022) and found stronger knockdown with DMG-PEG LNP versus their DSG-PEG LNP. One difference between DMG-PEG and DSG-PEG is the number of carbons in the hydrophobic tail (C14 versus C18, respectively), and others have demonstrated that a shorter tail results in more rapid dissociation of the PEGylated lipid and faster uptake into cells (Chen et al., 2014). However, faster cellular uptake may come at a cost for in vivo bone transfection, as LNPs may enter cells in other tissues that they are exposed to before bone, such as liver, reducing delivery to bone tissue (Basha et al., 2022). Similar to helper lipids, these findings suggest that future studies of LNP delivery of nucleic acid to bone must balance serum stability, endocytosis efficiency, and endosomal escape when selecting PEGylated lipids in LNP formulations.

Bone-targeting ligands are additional LNP formulation adjuncts included in this review, and three studies assessed in vitro nucleic acid transfection efficiency among LNPs with and without bone-targeting ligands. Liang (2015) compared siRNA transfection efficiency of their CH6 aptamer-LNP versus aptamer-less and random aptamer controls and found transfection with the CH6-LNP to be more efficient (Liang et al., 2015). Aptamers are nucleic acid strands with affinity for specific molecules, including cellular surface ligands, and the Cell-SELEX assay used by Liang is an established protocol for screening large libraries of aptamers for affinity to different cell types (Sefah et al., 2010). Liang's findings support Cell-SELEX being used as a method of rational design for LNP bone-targeting aptamers, and they also support the CH6 aptamer's use in future LNP formulations intended to target osteoblasts. Z. Li et al. (2025b) provide fewer details about the derivation of their mouse bone marrow MSC-targeting aptamer, but they also show it to have more efficient delivery of mRNA than an aptamer-less control. Thus, this aptamer could also be useful as a bone-targeting ligand for this cell type.

After demonstrating in vitro efficacy of their LNPs, all but one study evaluated the ability of the included LNPs to deliver nucleic acid to bone tissues in vivo. Despite the proposed challenges with in vivo transport of nucleic acids from injection site to bone tissue, seven LNPs were demonstrated to directly deliver nucleic acids to bone tissue after in vivo administration. One potential explanation for the in vivo efficacy of these LNPs is size. Based on our inclusion criteria, all LNPs included in this study had an average diameter that was no more than one standard deviation above 100 nm. 100 nm is a commonly targeted size maximum for LNPs being developed for clinical purposes (McMillan et al., 2024), and it also represents the upper limit of the diameter of bone marrow sinusoids (Wang et al., 2010), which has been cited as a limiting factor for LNP delivery of nucleic acid drugs to cells in compact bone (Marenzana and Arnett, 2013). Three studies went one step further by also showing specifically which bone cells were transfected in vivo, with Liang's CH6-LNP transfecting osteoblasts, the Basha 2016 LNP transfecting osteocytes, and the Basha 2022 DSG-PEG LNP transfecting MSCs. Thus, seven of the LNPs identified in our review could reasonably be used as starting formulations for novel bone-targeting LNPs with some being even better platforms for targeting specific bone cell types.

Given the wide variety of in vivo systems used for evaluating these LNPs, comparisons of the different LNP formulations' impact on in vivo bone delivery are challenging, but some comparisons can be made. Liang et al. (2015) and Xu et al.'s (2024) findings of improved nucleic acid delivery to bone using the CH6-LNP versus non-aptamer LNPs provides further support that osteoblast-specific aptamers should be strongly considered for incorporation into future bone-targeting LNPs. Importantly, the CH6-LNPs lower delivery to non-bone tissue types demonstrates it as a promising option for disease processes where off-target delivery may be deleterious as well. Given that the formulations of the Liang et al. (2015) and Xu et al. (2024) LNPs were quite different other than the presence of the CH6 aptamer, this suggests that the aptamer may be principally responsible for this efficacy and specificity. The other study that compared different lipidic formulations was Nelson et al. (2024) who found that their MC3 LNP and SM102 lipidic delivery vector displayed similar delivery in their local fracture injection model. One explanation for this could be the delivery model. As stated earlier, a key difference between these two formulations is their helper lipids, which are proposed to have different serum stability, a characteristic that could lead to more distant transit in the blood. However, this would not necessarily result in differences in a local treatment model, where the LNPs are delivered directly to bone. Therefore, each of these formulations could be equally as effective as starting points for LNPs being developed for local bone injection.

The last set of metrics reviewed in this study were in vitro toxicities and in vivo adverse effects. Seven studies evaluated in vitro cytotoxicity, and none reported any significant toxicity relative to negative controls. Interestingly, one of the two studies that did not report on toxicity was Xu's, which was also the only study using a cationic lipid instead of an ionizable lipid (Xu et al., 2024). Prior to the development of ionizable LNPs, cationic formulations were more common, but they were largely replaced by ionizable lipids due to the cytotoxicity associated with their positive charge (Mrksich et al., 2024). Thus, the promise of Xu et al.'s (2024) CH6-LNP in terms of efficacy and specificity may be lessened by an unfavorable toxicity profile, which needs further investigation. In vivo adverse effects were evaluated in six studies, and the only abnormal measures were reported by Nelson as elevated inflammatory markers (CRP and TNF-alpha) for MC3 LNPs versus PBS treatment (Nelson et al., 2024). Inflammatory responses are common in studies of LNP delivery of nucleic acids, including for FDA - approved treatments such as Onpattro and the two aforementioned COVID-19 vaccines (Moghimi and Simberg, 2022). However, other measures, such as weight loss and organ toxicity, were negative in all other studies, supporting the relative safety of LNPs as nucleic acid delivery vectors.

There are a number of limitations to this review. First, our search yielded a small number of studies and LNPs. Although we attribute this to the novel nature of this research and our strict inclusion criteria, it limits our ability to make conclusions about the ever-growing number of LNP formulations' capacity to target bone cells and tissues. Second, as evidenced by our analysis of quality and risk-of-bias, many of these studies lack details on important practices to ensure scientific rigor, such as controls, randomization, and blinding. Third, many different in vitro and in vivo systems were used to analyze delivery of these LNPs, making it challenging to make more comparisons with LNPs from different studies. Fourth, some studies of bone anabolism did not consider the endocrine/paracrine influences on bone anabolism, so changes in bone structure may not have been due to direct delivery of LNP cargo to bone in some cases. Fifth, although in vivo toxicity across studies was low, follow-up time was short in these analyses, so it is unclear if these LNPs have long-term adverse effects. Overall, higher-quality studies comparing more LNP formulations in similar models with longer-term toxicity evaluation will be valuable to help determine the optimal LNP formulations for delivery of nucleic acids to osteoblast-lineage cells and bone.

## 5 Conclusions

A variety of LNP formulations are effective for transfecting osteoblast-lineage cells with nucleic acids and for delivering nucleic acid to bone tissue, demonstrating the potential of LNPs for nucleic acid delivery as a treatment for bone disorders. Individual components of LNP formulations influence cell and tissue specificity, transfection efficiency, and LNP serum stability. Therefore, each component should be considered when formulating bone-targeting LNPs. Given their lack of in vitro toxicity and minor in vivo adverse effects limited to increased inflammatory markers, these bone-targeting ionizable LNPs should be considered safe for use in future studies.

## Funding support

This work was supported by funds from the UCSF Craniofacial Center. AM is supported by the 10.13039/100008069UCSF CIRM Scholars Training Program EDUC4-12812 fellowship. Research reported in this publication was also supported by the 10.13039/100000069National Institute of Arthritis and Musculoskeletal and Skin Diseases of the National Institutes of Health under Award Number T32AR080618. The content is solely the responsibility of the authors and does not necessarily represent the official views of the National Institutes of Health. The sponsors were not directly involved in design, collection, analysis, interpretation of the data, writing of the report, or submission of the article.

## CRediT authorship contribution statement

**Alexander I. Murphy:** Conceptualization, Data curation, Formal analysis, Funding acquisition, Investigation, Methodology, Visualization, Writing – original draft. **Christopher Vargas:** Conceptualization, Data curation, Formal analysis, Investigation, Methodology, Visualization, Writing – original draft. **Jason H. Pomerantz:** Conceptualization, Funding acquisition, Supervision, Writing – review & editing.

## Declaration of competing interest

The authors declare the following financial interests/personal relationships which may be considered as potential competing interests:

Jason H Pomerantz reports financial support was provided by UCSF Craniofacial Center. Alexander Murphy reports financial support was provided by National Institutes of Health. Alexander Murphy reports financial support was provided by California Institute for Regenerative Medicine. If there are other authors, they declare that they have no known competing financial interests or personal relationships that could have appeared to influence the work reported in this paper.

## Data Availability

Data will be made available on request.
